# A Comparison of Opioid Overdose Risk Behaviors by Race and Ethnicity Among Overdose Survivors in Boston, MA, and San Francisco, CA

**DOI:** 10.21203/rs.3.rs-8216462/v1

**Published:** 2026-02-13

**Authors:** Gabriela E Reed, Vanessa M McMahan, Yi-Shin Grace Chang, Natrina L Johnson, Alexander Y. Walley, Phillip O Coffin, Miriam TH Harris

**Affiliations:** University of North Carolina at Chapel Hill; Harvard Medical School, Harvard Pilgrim Health Care Institute; San Francisco Department of Public Health; Boston University; Boston University; San Francisco Department of Public Health; Boston University

**Keywords:** overdose, race, ethnicity, risk behaviors, drug use

## Abstract

**Background:**

Black and Latine people are at high risk for opioid overdose mortality. Interventions focused on overdose risk behaviors may offer opportunities to reduce the risk of overdose, but little research has addressed racial and ethnic differences in risk behaviors. We explored differences in risk behaviors among Black, Latine, and White individuals who used opioids and had a history of prior overdose.

**Methods:**

We used data from REBOOT 2.0 (the REpeated dose Behavioral intervention to reduce Opioid Overdose Trial) based in Boston, MA, and San Francisco, CA, which enrolled people with opioid use disorder and a history of opioid overdose. We used the Andersen behavioral model to inform covariate selection for a multivariable logistic regression analysis examining associations between race/ethnicity (Black, Latine, and White) and three opioid overdose risk behaviors (using opioids alone, not using a tester dose, and using alcohol or benzodiazepines on the same day as opioids).

**Results:**

Of 247 participants, 19% were Black, 16% were Latine and 66% were White. More than 20% in each group reported each risk behavior. In unadjusted analyses, Black participants had higher odds of using alone (OR=2.17, 95% CI 1.08–4.36) but lower odds of not using a tester dose (OR=0.48, 95% CI 0.24–0.93) compared to White participants. After adjusting for age, gender and structural determinants of health (e.g., housing, incarceration, education, opioid use disorder treatment) the adjusted odds of Black participants using alone (aOR=2.05, 95% CI 0.99–4.26) and using a tester dose (aOR=0.56, 95% CI 0.28–1.13) were no longer statistically significant.

**Conclusions:**

Overdose risk behaviors were common among Black, Latine, and White overdose survivors in San Francisco and Boston. Overdose risk behaviors were not significantly different for Black or Latine compared to White overdose survivors after adjusting for age, gender, and structural determinants of health. Overdose risk reduction strategies for those at the highest risk for overdose death need to move beyond risk behavior reduction education and address structural determinants of health.

**Trial registration:**

Details found at clinicaltrials.gov, NCT02093559.

## Background

While the overall age-adjusted rate of drug overdose deaths in the United States decreased by 4.0% between 2022 and 2023, the rate increased among Black people from 47.5 to 48.9 deaths per 100,000 and remained the same among Latine people[1] at 22.7 to 22.8 deaths per 100,000 (2). Thus, Black and Latine people remain disproportionately impacted by the overdose crisis (3). Interventions focused on overdose risk behaviors may offer opportunities to reduce the risk of overdose and overdose fatality for non-Latine Black and Latine individuals. Risk behaviors that increase the risk of overdose include using opioids with other sedating substances, such as alcohol and/or benzodiazepines. Behaviors that can increase the chance of fatality from overdose include using opioids alone, reducing the chance of a bystander being able to respond and intervene in an overdose event. Conversely, protective behaviors include using a tester dose or small amount of opioid to “test” the drug being used (4–6).

There is a paucity of evidence and inconsistent results from prior studies examining racial and ethnic differences in opioid overdose risk behaviors. A study of 196 participants across three US cities found that when fentanyl was suspected in their drug supply, Black and Latine participants were twice as likely as White participants to engage in protective overdose behaviors, such as using smaller amounts, taking turns using drugs, or obtaining naloxone (7). However, these behaviors were analyzed collectively, not individually (7). Another study, examining 20 years of death records, showed that Black individuals had higher rates of combined alcohol and opioid-related deaths, while Latine individuals had lower rates of these deaths, compared to White individuals (8). Among 509 individuals from Rhode Island, regular use of benzodiazepines was significantly higher among White participants compared to other racial and ethnic groups (9). This study did not find other variations in drug use behaviors or harm reduction practices by race or ethnicity (9). Given the inconclusive and limited nature of the existing literature, a more nuanced understanding of individual-level overdose risk behaviors is needed to address overdose inequities among racial and ethnic populations and inform tailored overdose prevention interventions.

To address this gap, this study explored differences in overdose risk behaviors among Black, Latine, and White individuals at high risk of overdose with a history of opioid use, previous overdose, and naloxone receipt. Understanding overdose risk behaviors among those with a history of overdose is particularly important because overdose survivors are at high risk of subsequent fatal overdose (10–12).

## Methods

### Study design and population

Data were derived from the REBOOT (REpeated dose Behavioral intervention to reduce Opioid Overdose) 2.0 study. Details of the REBOOT study design have been described elsewhere (13,14). Briefly, REBOOT was a two-site randomized-controlled trial designed to assess the effectiveness of a repeated counseling intervention to reduce opioid overdose. The study was approved by the University of California, San Francisco Institutional Review Board (#17–24203).

Adults, aged 18 to 65 years old, from Boston, MA, and San Francisco, CA, were consented and enrolled between April 2019 and June 2022. All participants had moderate to severe opioid use disorder per the Diagnostic and Statistical Manual of Mental Disorders, Fifth Edition; self-reported past 14-day non-prescribed opioid use, an opioid overdose in the past three years, and prior receipt of take-home naloxone; and provided an opioid-positive urine drug screen (or third-party confirmation of opioid use for remote visits during COVID-19 restrictions). Data were collected at baseline as well as every four months over the 16-month study duration, either in-person or via telephone, and entered into REDCap by research staff (15,16). The study questionnaire included demographic data, substance use and overdose risk behaviors, and substance use disorder treatment experiences. Participants were compensated on an escalating scale between $20 (e.g., screening visit) and $100 (final study visit) for study participation. In this study, we analyzed data from the REBOOT 2.0 enrollment visit and included trial participants who self-identified as Black, Latine, or White.

### Outcome variables

We selected three opioid overdose risk behaviors informed by the literature that can increase risk of overdose occurrence or fatality as our primary outcome measures (4,17–20): 1) using alone, defined as using opioids without someone else at the same time, 2) not using a tester dose, defined as not using a smaller amount first or not using slowly to test the strength of one’s drugs, and 3) using opioids the same day as using alcohol and/or benzodiazepines. We did not explore naloxone use because the trial required previous naloxone receipt for enrollment, which could bias results.

We combined co-use of alcohol and benzodiazepines as one of our primary outcome measures because both increase the risk of respiratory depression and sedation when used with opioids. However, existing literature indicates differences in co-use rates among Black and Latine individuals for alcohol versus benzodiazepines with opioids (8,9,21). Combining the co-use of alcohol and/or benzodiazepines with opioids may obscure these racial differences. Therefore, we also analyzed the separate use of alcohol and benzodiazepines on the same day as opioid use to understand the contribution of each individual substance.

All behaviors were assessed over the preceding four months. How often individuals engaged in a risk behavior was assessed using a Likert scale of “never,” “rarely,” “often,” “sometimes,” or “always” when using opioids. For this analysis, behaviors were dichotomized into not engaged defined as “never” or “rarely” versus engaged defined as “sometimes,” “often,” or “always.” Outcomes were presented as the responses associated with increased overdose risk. For using alone and using alcohol or benzodiazepines on the same day as using opioids, sometimes, often, or always, were considered to increase overdose risk. For using a tester dose, never or rarely were considered to increase risk.

### Predictors

The independent variables were self-reported Black vs. White race and Latine vs. non-Latine ethnicity. Options for racial and ethnic identification included Native American/Alaska Native, Asian, Native Hawaiian or other Pacific Islander, Black or African American, White, More than one race, or Other and Hispanic/Latinx or non Hispanic/Latinx. Latine included any participants reporting ethnicity as Hispanic/Latinx, regardless of race. Black and White included participants reporting Black (African American, Black, inclusive of multiracial individuals) or White race, respectively, and not identifying as Hispanic/Latinx ethnicity. We excluded individuals who did not identify as Black, White, or Hispanic/Latinx.

### Descriptive variables

Sociodemographic characteristics included age, gender (male or trans male, female or trans female), housing (stable, unstable), education (less vs. more than receipt of high school diploma or equivalent), incarceration in the previous four months, baseline HIV status, health insurance (private, public or Veterans Affairs, or uninsured), employment status defined as having a full or part-time job, and site of participation (Boston, San Francisco). Substance use behaviors included frequency (reported as number of days out of past 30; categorized into daily use vs some use vs no use in the past 30 days) and primary route (injection, smoking, nasal, oral) of opioid use (defined as use of heroin, non-prescribed fentanyl, or other non-prescribed opioids/painkillers) and the frequency of stimulant use in the past 30 days (defined as use of cocaine or methamphetamine/amphetamines).

We also reported use of fentanyl test strips (never or rarely vs sometimes, often, or always) and buying opioids from a dealer you do not regularly go to for opioids (never or rarely vs sometimes, often, or always) over the past four months to provide details on additional overdose risk behaviors that we did not conduct comparisons for because they were less well established in the literature. Substance use disorder treatment characteristics included the use of inpatient treatment (being in a residential treatment program, inpatient detox program, or sober house) and outpatient interventions (attending AA/NA meetings or receiving outpatient counseling for substance use) in the past four months.

### Conceptual Framework

To understand how individual health behaviors may be impacted by race or ethnicity, it is critical to consider their structural context, specifically racism and the related inequities in social determinants of health and healthcare access (9,22,23). Thus, we applied the Andersen behavioral model, a health equity framework, to account for structural racism in our exploration of individual overdose risk behaviors (24–26). This model posits that individual deleterious or protective health behaviors are predicated upon a sequence of predisposing, enabling, and need factors. Predisposing factors may include demographic characteristics such as age and gender (27–29). Enabling factors influence health service engagement, and include structural determinants such as housing, education, incarceration, insurance, and access to treatment (11,30–32). Lastly, need factors impact the necessity for a health behavior; for example, current use of non-prescribed opioids influences the need for protective overdose behaviors. We explored whether overdose risk behaviors varied between Black, Latine, and White participants, in the context of predisposing (age and gender) and enabling (medication for opioid use disorder [MOUD], research site, unstable housing, prior incarceration, and receipt of high school diploma or equivalent) factors among a group of overdose survivors, with a need factor for overdose risk reduction (see Table 2 for greater detail on how variables were measured and operationalized).

### Statistical analyses

We used descriptive statistics to summarize data with counts and percentages for categorical variables and measures of central tendency for continuous variables. Since continuous data were skewed, we used medians and interquartile ranges.

For each outcome, models were built using the following steps based on the Andersen behavioral model’s conceptual framework. First, bivariate logistic regression analyses were used to assess the association of race, ethnicity, predisposing, and enabling factors and each outcome of using alone, not using a tester dose, and using alcohol and/or benzodiazepines on the same day as opioids. Next, adjusted multivariable logistic regression analyses were performed to assess the association of race and ethnicity with the three overdose risk behaviors. Model 1 included variables measuring predisposing factors (the demographic covariates of age and gender). Model 2 added enabling factors (research site, MOUD treatment, housing, incarceration, and receipt of high school diploma or equivalent) to assess how these affected the association between race and ethnicity and the three overdose risk behavior outcomes. Lastly, we secondarily examined using alcohol or benzodiazepines on the same day as opioids separately to assess differences by race/ethnicity with the use of each of these substances independently. The significance level for all analyses was set at 0.05 and all analyses were performed using Stata 17 statistical software (StataCorp, College Station, TX).

## Results

Of 268 REBOOT participants, 247 were included in this analysis (21 did not identify as Black, Latine, or White); 66% were White, 19% Black, and 16% Latine (see Table 1). The median age was 42 years (IQR: 34–50) and 62% identified as male or trans male. At baseline, 64% were experiencing unstable housing, 10% had been incarcerated over the past four months, and 85% were unemployed. All participants reported non-prescribed opioid use in the past 30 days, of whom 54% reported daily use and 68% reported primary use by injection. Most participants used stimulants in the past 30 days (93%), of whom 35% reported daily use. Most (70%) had received MOUD in the past four months, 53% had engaged in outpatient recovery counseling, and 23% had undergone residential substance use disorder treatment. Most reported using alcohol or benzodiazepines the same day as opioids (57%), 52% did not use a tester dose, and 27% had used alone in the past four months.

### Bivariate Results

Table 2 presents the bivariate results. Black participants had higher odds of using alone (OR = 2.17, 95% CI 1.08–4.36, p = 0.03) but lower odds of not using a tester dose (OR = 0.48, 95% CI 0.24–0.93, p = 0.03), compared to White participants. For Latine compared to White participants, the association estimates with using alone (OR = 1.33, 95% CI 0.60–2.92, p = 0.48) and not using a tester dose (OR = 0.64, 95% CI 0.32–1.28, p = 0.21) were in the same direction as Black participants, but were not statistically significant. There were no statistically significant differences for using alcohol or benzodiazepines on the same day as opioids across race/ethnicity groups. Of the predisposing and enabling factors considered, being a participant from San Francisco was associated with lower odds of using alcohol or benzodiazepines on the same day as opioids (OR=0.45, 95% CI: 0.27–0.77, p <0.01).

### Multivariable Results: Model 1 (Predisposing factors only)

After adjusting for predisposing factors (Table 3), the associations of Black race with using alone (aOR = 2.01, 95% CI 0.99–4.10, p = 0.05) and not using a tester dose (aOR = 0.51, 95% CI 0.26–1.01, p = 0.05) compared to White race were attenuated, and no longer statistically significant (Table 3). Other analyses remained statistically insignificant.

### Multivariable Results: Model 2 (Predisposing and enabling factors)

In model 2 (Table 3), after adjusting for predisposing and enabling factors there were no statistically significant associations between race or ethnicity and overdose risk behaviors. See supplemental tables 1–3, 4a, and 4b for the full models encompassing all multivariate associations among the independent variables, predisposing and enabling factors, and the outcomes.

### Secondary Analyses

In multivariable analyses (Table 4) that separately examined the outcome of using alcohol or benzodiazepines with opioids separately, Black participants had lower odds of using benzodiazepines on the same day that they used opioids compared to White participants in both the models that adjusted only for predisposing factors (aOR = 0.34, 95% CI 0.16–0.75, p < 0.01) and for both predisposing and enabling factors (aOR = 0.34, 95% CI 0.15–0.76, p < 0.01). All other comparisons were not statistically significant.

## Discussion

In this study of participants with opioid use disorder and a history of overdose, using alcohol and benzodiazepines on the same day as opioids, not using a tester dose, and using alone were common overdose risk behaviors. Significant differences found in unadjusted analyses comparing Black and White overdose survivors, where Black participants had higher odds than White participants to use a tester dose and to use alone, were not significant after adjusting for age, gender, and structural determinants of health, specifically housing, incarceration, education, and MOUD treatment. Black participants had lower adjusted odds of using benzodiazepines compared to White participants in the adjusted secondary analysis.

The association observed between Black compared to White race and using alone (OR = 2.17, 95% CI 1.08–4.36) and not using a tester dose (OR = 0.48, 95% CI 0.24–0.93) in the unadjusted analyses reflected different frequencies of overdose prevention behaviors. Our study was a secondary analysis of data from an interventional cohort study of people who all had a history of overdose. The fact that all participants had overdose experiences suggests that both the burden of overdose risk and the use of protective behaviors were substantively enhanced within each of the racial and ethnic groups (7,33). After adjusting for housing, incarceration, education, and MOUD treatment, which are manifestations of structural racism and also commonly experienced by all racial and ethnic groups in this study, the findings were no longer statistically significant. The varying unadjusted frequencies of overdose risk behaviors by race may reflect different strategies for coping with the structural determinants of health faced by overdose survivors who continue to use opioids. For example, social and economic marginalization may limit Black people who use drugs from accessing communal drug-use spaces, while fears of disproportionate surveillance and policing may further reinforce their decision to use drugs alone (34). Thus, Black overdose survivors who use drugs alone may adapt by testing their drug doses more frequently to reduce the risk of overdose. Our findings suggest efforts to address overdose risk for people who use drugs need to move beyond risk reduction education and incorporate social justice approaches that address structural determinants of health, such as housing, incarceration, and access to MOUD (35).

We did not detect statistically significant differences by Latine ethnicity for any of the risk behaviors. Future studies should continue to explore opioid overdose risk behaviors among Latine communities in greater detail, including through recruitment and selection of protocols that facilitate Latine engagement in research. Such strategies might include greater engagement of community organizations that serve Latine populations, as well as bicultural and bilingual research staff who can facilitate rapport-building with monolingual Spanish speakers and/or those who prefer to communicate in Spanish (36–39).

We found Black participants to have lower odds of using benzodiazepines on the same day that they used opioids. However, that finding may have been counterbalanced by possibly higher odds of using alcohol on the same day as opioids; although that finding was not significant, prior investigators have found higher rates of deaths attributed to alcohol and opioids together among Black people.(8) Given the similar pharmacology of benzodiazepines and alcohol, and the similar risk of using those drugs with opioids, we combined those two classes for analysis, which may have canceled out differences in substance use. Black patients are less likely to receive prescriptions for benzodiazepines,(40,41) an example of racial bias in medical care that might be paradoxically protective by reducing co-use with opioids; nonetheless, our data suggest that patients may instead find another means to obtain pharmacologic sedation. Though providers often counsel about the risks of alcohol and benzodiazepine use with opioids, our analyses suggest that substance-specific recommendations may be needed, and future research with Black people who report using alcohol and opioids should inform these recommendations.

This study must be interpreted in the context of several limitations. First, our assessment of engagement in risk behaviors was based on self-report data and is therefore subject to recall and social desirability biases. We conducted a cross-sectional analysis, so risk behaviors could have preceded outcomes that were also measured over the last four months. Next, the exclusion of monolingual Spanish-speaking participants may have biased our results comparing behaviors among Latine to White participants toward the null, as data has shown that numerous health outcomes are worse among Spanish-speaking compared to English-speaking Latine people (46). Similarly, overdose risk varies substantially by specific Latine ethnic identities (47). These differences may be obscured when Latine ethnicities are aggregated as we did in this study. In general, the REBOOT study was not designed to detect differences by race or ethnicity, thus the recruitment strategy and sample size may have contributed to the null findings. In future studies examining racial and/or ethnic variation in substance use risk behaviors, adequate statistical power and tailored recruitment strategies are warranted. Furthermore, this study drew participants from the REBOOT 2.0 cohort, which was based in Boston and San Francisco, two urban cities that are relatively well-resourced in terms of harm reduction services, and eligibility for the study required prior naloxone receipt. Thus, participants may have been more likely to engage in protective behaviors than others, such as those living in rural areas (48), those who have not used naloxone, or those who are not willing to participate in research (49). Also, this analysis focused on identifying racial and ethnic differences using three mutually exclusive racial/ethnic groups; we excluded participants who did not self-identify with these groups. We did not explore differences in risk behaviors among American Indian and Alaskan Native people due to the low enrollment of individuals from these groups in the parent study. Future overdose risk studies should be designed to focus on these underrepresented groups given their high rates of opioid-related deaths (50).

## Conclusions

After adjusting for structural determinants in a cohort of people who had survived an opioid overdose, we did not identify differences in opioid overdose risk behaviors among Black or Latine participants compared to White participants. Addressing structural barriers related to housing and access to services that disproportionately affect Black and Hispanic/Latine people, may be important for mitigating known racial and ethnic disparities in overdose risk.

## Supplementary Material

Supplementary Files

This is a list of supplementary files associated with this preprint. Click to download.

- riskbehaviorssupplementforcirculation6.1.25.docx

## Figures and Tables

**Table 1.** Baseline characteristics of participants with a history of opioid overdose from Boston and San Francisco, 2019 – 2022 (N = 247*).

	Total n (%)	White participants n (%)	Black participants n (%)	Latine participants n (%)
Total	247 (100)	162 (66)	46 (19)	39 (16)
Age – [median, IQR]	42 (34–50)	42 (34–49)	47 (38–56)	39 (35–49)
Gender				
Male or trans male	153 (62)	100 (62)	26 (57)	27 (69)
Female or trans female	94 (38)	62 (38)	20 (43)	12 (31)
Site				
San Francisco	142 (57)	96 (59)	23 (50)	23 (59)
Boston	105 (43)	66 (41)	23 (50)	16 (41)
Treated with MOUD^a,b^	172 (70)	118 (73)	30 (65)	24 (62)
Unstably housed^a,c^	158 (64)	104 (64)	29 (63)	25 (64)
Incarcerated^a,d^	25 (10)	15 (9)	4 (9)	6 (15)
Completed high school/GED or above	217 (88)	147 (91)	43 (93)	27 (69)
HIV Positive^e^	18 (7)	10 (6)	6 (13)	2 (5)
Currently insured	245 (99)	160 (99)	46 (100)	39 (100)
Currently employed	38 (15)	23 (14)	8 (17)	7 (18)
Past 30-day cocaine or methamphetamine use				
Daily use	80 (32)	50 (31)	16 (35)	14 (36)
Some use	150 (61)	104 (64)	23 (50)	23 (59)
No use	17 (7)	8 (5)	7 (15)	2 (5)
Past 30-day opioid use^f^				
Daily use	134 (54)	79 (49)	28 (61)	27 (69)
Some use	113 (46)	83 (51)	18 (39)	12 (31)
No use	0 (0)	0 (0)	0 (0)	0 (0)
Primary route of opioid use^a,f^				
Inject	167 (68)	116 (72)	24 (52)	27 (69)
Smoke	43 (17)	31 (19)	6 (13)	6 (15)
Sniff	36 (15)	14 (9)	16 (35)	6 (15)
Oral	1 (0)	1 (1)	0 (0)	0 (0)
Used fentanyl test strips^g^	32 (13)	19 (12)	9 (20)	4 (10)
Purchased opioids from a new dealer^g^	103 (42)	71 (44)	15 (33)	17 (44)
Residential treatment^a,h^	58 (23)	43 (27)	9 (20)	6 (15)
Engaged in outpatient recovery counseling^a,i^	131 (53)	85 (52)	27 (59)	19 (49)
Used alcohol and/or benzodiazepines on the same day as opioids^a,g,l^	142 (57)	95 (59)	26 (57)	21 (54)
Did not use a tester dose^a,j^	129 (52)	93 (57)	18 (39)	18 (46)
Used alone^a,k^	66 (27)	37 (23)	18 (39)	11 (28)
Used alcohol and opioids on the same day^a,g^	72 (29)	93 (57)	19 (41)	9 (23)
Used benzodiazepines and opioids on the same day^a,g^	104 (42)	37 (23)	10 (21)	16 (41)

* 21 individuals from the original study cohort were excluded.

a Variable assessed over the past four months.

b MOUD defined as taking methadone from a methadone clinic for OUD, taking Suboxone prescribed by a doctor, receiving Sublocade injection by a doctor, receiving Vivitrol injection by a doctor, or taking naltrexone prescribed by a doctor.

c Unstable housing operationalized as spending any nights on the street or in a homeless shelter.

d Incarceration defined by self-report.

e Two individuals had unknown HIV status.

f Reported as a combined hierarchical variable of fentanyl, heroin, and other opioids.

g Operationalized as response of “sometimes,” “often,” or “always” to behavior.

h Residential treatment is attendance in a residential treatment program, inpatient detox program, or sober house.

i Outpatient recovery counseling is attendance of AA/NA meetings or receipt of outpatient counseling for substance use.

j Operationalized as response of “never” or “rarely” to behavior of using a tester dose or slow shot.

k Operationalized as response of “never” or “rarely” to behavior of using with someone else at the same time.

l One missing response.

**Table 2.** Bivariate association of baseline characteristics of participants with a history of opioid overdose from Boston and San Francisco with overdose risk behaviors of using alcohol or benzodiazepines on the same day as opioids, not using a tester dose, and using alone, 2019 – 2022 (N = 247).

	Used alcohol or benzodiazepines the same day as opioids OR (95%CI) p-value	Did not use a tester dose OR (95%CI) p-value	Used alone OR (95%CI) p-value
**Independent Variables**			
Race & Ethnicity			
White	Ref	Ref	Ref
Black	0.90 (0.47 – 1.75) 0.76	0.48 (0.24 – 0.93) 0.03	2.17 (1.08 – 4.36) 0.03
Latine	0.81 (0.40 – 1.64) 0.56	0.64 (0.32 – 1.28) 0.21	1.33 (0.60 – 2.92) 0.48
**Predisposing Variables**			
Age for every 10-year increase [median, IQR]	0.94 (0.73 – 1.21) 0.61	0.80 (0.62 – 1.03) 0.08	1.20 (0.91 – 1.60) 0.19
Gender			
Male or trans male	Ref	Ref	Ref
Female or trans female	1.02 (0.61 – 1.72) 0.93	1.22 (0.73 – 2.05) 0.45	1.40 (0.79 – 2.48) 0.25
**Enabling Variables**			
Site			
Boston	Ref	Ref	Ref
San Francisco	0.45 (0.27 – 0.77) <0.01	1.38 (0.83 – 2.29) 0.21	0.78 (0.44 – 1.38) 0.39
Treated with MOUD^a,b^	1.45 (0.84 – 2.51) 0.19	1.49 (0.86 – 2.57) 0.15	1.23 (0.66 – 2.29) 0.52
Unstably housed^a,c^	1.32 (0.78 – 2.23) 0.31	1.37 (0.81 – 2.31) 0.24	0.63 (0.36 – 1.13) 0.12
Incarcerated^a,d^	0.54 (0.23 – 1.24) 0.15	1.18 (0.52 – 2.72) 0.69	0.49 (0.16 – 1.49) 0.21
Obtained GED or above	1.05 (0.49 – 2.27) 0.90	0.95 (0.44 – 2.04) 0.90	0.83 (0.36 – 1.92) 0.67

a Variable assessed over the past four months.

b MOUD defined as taking methadone from a methadone clinic for OUD, taking Suboxone prescribed by a doctor, receiving Sublocade injection by a doctor, receiving Vivitrol injection by a doctor, or taking naltrexone prescribed by a doctor.

c Unstable housing operationalized as spending any nights on the street or in a homeless shelter.

d Incarceration defined by self-report.

**Table 3.** Adjusted multivariable association of race and ethnicity among participants with a history of opioid overdose from Boston and San Francisco with overdose risk behaviors of using alcohol or benzodiazepines on the same day as opioids, not using a tester dose, and using alone, 2019 – 2022 (N = 247).

	Used alcohol or benzodiazepines the same day as opioids aOR (95%CI) p-value	Did not use a tester dose aOR (95%CI) p-value	Used alone aOR (95%CI) p-value
**Model 1:** Predisposing factors^a^			
White	Ref	Ref	Ref
Black	0.93 (0.47 – 1.82) 0.83	0.51 (0.26 – 1.01) 0.05	2.01 (0.99 – 4.10) 0.05
Latine	0.81 (0.40 – 1.64) 0.55	0.63 (0.31 – 1.29) 0.21	1.37 (0.62 – 3.04) 0.43
**Model 2:** Predisposing & enabling factors b			
White	Ref	Ref	Ref
Black	0.89 (0.44 – 1.78) 0.74	0.56 (0.28 – 1.13) 0.11	2.05 (0.99 – 4.26) 0.05
Latine	0.85 (0.40 – 1.81) 0.68	0.65 (0.31 – 1.37) 0.26	1.40 (0.61 – 3.23) 0.43

[a] Model 1 adjusted for age and gender.

b Model 2 adjusted for age, gender, site, MOUD treatment, housing, incarceration, and GED receipt.

**Table 4.** Bivariate and adjusted multivariable association of race and ethnicity among participants with a history of opioid overdose from Boston and San Francisco with overdose risk behaviors of using alcohol on the same day as opioids and using benzodiazepines on the same day as opioids, 2019 – 2022 (N = 247).

	Used alcohol on the same day as opioids aOR (95%CI), p-value	Used benzodiazepines on the same day as opioids aOR (95%CI), p-value
**Bivariate association**		
White	Ref	Ref
Black	1.87 (0.95 – 3.70) 0.07	0.30 (0.14 – 0.64) <0.01
Latine	0.80 (0.35 – 1.81) 0.59	0.75 (0.37 – 1.52) 0.42
**Model 1:** Predisposing factors [a]		
White	Ref	Ref
Black	1.60 (0.79 – 3.24) 0.19	0.34 (0.16 – 0.75) <0.01
Latine	0.80 (0.35 – 1.84) 0.60	0.72 (0.35 – 1.48) 0.37
**Model 2:** Predisposing and enabling factors [b]		
White	Ref	Ref
Black	1.63 (0.80 – 3.35) 0.18	0.34 (0.15 – 0.76) <0.01
Latine	0.72 (0.30 – 1.74) 0.47	0.72 (0.33 – 1.56) 0.41

a Model 1 adjusted for age and gender.

b Model 2 adjusted for age, gender, site, MOUD treatment, housing, incarceration, and GED receipt.

## Footnotes

[1] In this paper, we use the term Black to refer to non-Latine Black and/or African American individuals and the term White to refer to non-Latine White individuals. We use the gender-neutral term Latine to refer to Hispanic and/or Latino/a/e/x individuals (1).

## Abbreviations

AA/NA: Alcoholics Anonymous / Narcotics Anonymous
BMHSU: Behavioral Model of Health Services Use
GED: General Education Diploma
HIV: Human Immunodeficiency Virus
MOUD: Medications for opioid use disorder
OUD: Opioid use disorder
REBOOT 2.0 Study: REpeated dose Behavioral intervention to reduce Opioid Overdose
